# Efficient Photocatalytic Degradation of Malachite Green in Seawater by the Hybrid of Zinc-Oxide Nanorods Grown on Three-Dimensional (3D) Reduced Graphene Oxide(RGO)/Ni Foam

**DOI:** 10.3390/ma11061004

**Published:** 2018-06-13

**Authors:** Qing Wang, Chaoyue Cai, Mingyan Wang, Qian Guo, Biao Wang, Weina Luo, Yujuan Wang, Chenyan Zhang, Lihua Zhou, Dongen Zhang, Zhiwei Tong, Yuqing Liu, Jun Chen

**Affiliations:** 1 Department of Chemical Engineering, Huaihai Institute of Technology, Lianyungang 222005, China; 15261392025@163.com (Q.W.); chaoyuecai2018@163.com (C.C.); guoqian20180707@163.com (Q.G.); wangbiao20180509@outlook.com (B.W.); weinaluo201801010@163.com (W.L.); luoweina0011@hotmail.com (Y.W.); chengyanzhang@outlook.com (C.Z.); y2005zlh@163.com (L.Z.); zdewxm@aliyun.com (D.Z.); tongzw@hhit.edu.cn (Z.T.); 2 Intelligent Polymer Research Institute, ARC Centre of Excellence for Electromaterials Science, Australian Institute of Innovative Materials, University of Wollongong, Northfields Avenue, Wollongong, NSW 2522, Australia; yl037@uowmail.edu.cn

**Keywords:** reduced graphene oxide, ZnO nanorods, Ni foam, photocatalyst, organic dye degradation

## Abstract

A hybrid of ZnO nanorods grown onto three-dimensional (3D) reduced graphene oxide (RGO)@Ni foam (ZnO/RGO@NF) is synthesized by a facile hydrothermal method. The as-prepared hybrid material is physically characterized by SEM, XRD, Raman, and X-ray photoelectron spectroscopy (XPS). When the as-prepared 3D hybrid is investigated as a photocatalyst, it demonstrates significant high photocatalytic activity for the degradation of methylene blue (MB), rhodamine (RhB), and mixed MB/RhB as organic dye pollutants. In addition, the practical application and the durability of the as-prepared catalyst to degradation of malachite green (MG) in seawater are firstly assessed in a continuous flow system. The catalyst shows a high degradation efficiency and stable photocatalytic activity for 5 h continuous operation, which should be a promising catalyst for the degradation of organic dyes in seawater.

## 1. Introduction

Malachite green (MG) is a common triphenylmethane compound that was originally developed as a dye agent in leather tanning, textile dyeing and hair colorings, which was introduced as an ectoparasiticide, fungicide and antiseptic agent in aquaculture in 1933 [1,2]. Indeed, MG has been extensively used in fisheries for many decades due to its low cost, ready availability, and high efficacy for the treatment or prevention of external fungal and parasitic infections in fish [2,3]. But later, it has been found to be toxic and tumor promoter [4,5,6,7,8,9]. However, the accumulation of this dye in fish or shrimp could cause genotoxic, carcinogenic, and mutagenic harms to human through the food chain [5,6,7,8]. However, despite these regulations, there have been numerous reports concerning MG contamination of culture fish for food purpose owing to illegal use of MG in fish farms [10,11,12]. The fish ponds are drained periodically in order to facilitate the fish harvest process and it is common practice to discharge the pond sediment and the waste water to the environment. These activities occur throughout the year and result in seawater contamination. A recent study demonstrated that the wild European eel distributed in various aquatic environments in Belgium also contained MG in their muscle tissue [13]. Therefore, the efficient treatment and removal of the MG residues in seawater is of the most urgent and important problem.

The photocatalysis, a ‘green approach’, is at the forefront of fundamental research to complete mineralization of organic dyes in wastewater. Researchers have already developed numerous materials as the photocatalysts, and the earliest study is based on the TiO_2_ semiconductor material [14] and its modification [15]. Meanwhile, various novel photocatalytic materials have emerged, such as nano zinc oxide (ZnO) [16,17], CdS [18], 2D PbMoO_4_ [19,20], g-C_3_N_4_ [21,22], and their composites [23], etc. Among the photocatalysts, ZnO has been extensively used over years due to its attractive properties, such as the low cost, non-toxicity, and photo sensitivity [24,25]. Unfortunately, the high recombination rate of the photogenerated e^−^ and h^+^ pairs makes ZnO inefficient for photocatalytic activities. In addition, the wide band gap also hinders its practical application in the visible-light region of solar energy. Moreover, nanometer scale ZnO is easy to agglomerate in suspension. Therefore, assembling composite materials consisting of ZnO nanocrystals and functional nanomaterials would be a better way to solve these issues.

As a rising star of the carbon family, reduced graphene oxide (RGO) has been widely used to design photocatalyst systems because of its excellent electron transfer properties, which can decrease the recombination of photoexcited charge carriers in metal oxides [26,27]. RGO can also narrow the band gap and prevent nanoparticle agglomeration by providing a good dispersion of metal oxides within the composites, and thus enhance the photocatalytic efficiency [28]. Omotayo A. Arotiba et al. synthesized a composite of silver (Ag), zinc oxide and RGO for efficient photoelectrochemical degradation and mineralization of organic pollutants in water treatment [29]. A quaternary TiO_2_/ZnO/RGO/Ag nanocomposite synthesized via facile microwave irradiation exhibited enhanced photoactivity for the degradation of rhodamine B under visible light [30]. Tingting Xu reported that a new ternary system consisting of ZnO nanorod/reduced graphene oxide (RGO)/CuInS_2_ quantum dots were prepared for photocatalytic application under visible light irradiation, and the hybrid achieved a greatly enhanced light absorption and charge transfer than that of the pristine ZnO [31]. However, in these investigations, the aqueous suspension of the catalyst nanoparticles has been used, the separation and recycling of the ultrafine nanocatalyst from the treated liquid are time-consuming and expensive in practical process. To solve the above problem, photocatalyst particles need to be immobilized onto a solid substrate. Huang et al. [32] integrate a flexible and porous organic matrix polyurethane foam (PUF) with nanosheet array like bismuth oxyhalides (BiOX) as efficient photocatalysts and the prepared BiOX/PUF foam showed a promoted photocatalytic performance for treating multiform organic contaminants, easy recovery, and good recycling. Chi-Jung Chang et al. [33] reported a photocatalyst that was synthesized by grown S-doped ZnO nanorods on stainless steel mesh, and high photocatalytic activity was obtained due to the increased surface area of the hierarchical photocatalyst. A novel photocatalyst that was based on TiO_2_–PANI composite supported on small pieces of cork has been reported and the prepared photocatalyst showed high efficiency for the degradation of methyl orange dye and other organic pollutants under solar irradiation [34]. Obviously, the immobilization of nano photocatalysts on various substrates is a convenient method to alleviate the problems of powder filtration, and catalyst recovery. It offers flexibility in photocatalyst handling for a water purification system for large scale practical application.

**Scheme 1:**
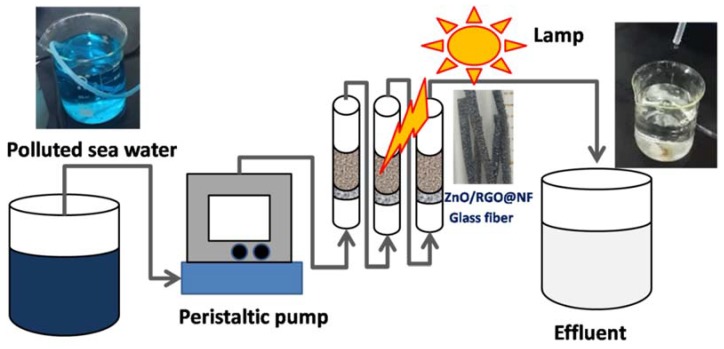
The continuous flow system for degradation of Malachite Green in Seawater.

In our previous work, we reported a facilely prepared three-dimensional (3D) RGO modified nickel foam (RGO@NF), which can be used as an ideal solid substrate to provide photocatalysts with high specific surface and good electronic transport capability [35]. In this work, ZnO nanorods were grown on this 3D RGO@NF template by a facile hydrothermal method (ZnO/RGO@NF). By this way, the ZnO nanorods and RGO were integrated on Ni Foam template, which facilitated the recycling of photocatalyst and made possible the handling for water purification system in the large scale. Used methylene blue (MB), rhodamine (RhB), and mixed MB and RhB solution as model compounds of organic pollution, the photocatalytic performance of the as-prepared hybrids was characterized under UV light irradiation. When compared with ZnO@NF and RGO@NF, significantly high photocatalytic degradation efficiency of the ZnO/RGO@NF was observed for these dyes, demonstrating its potential to be used as a catalyst in practice application. The practical application of the as-prepared catalyst for the degradation of MG in seawater was firstly examined in a continuous flow system (Scheme 1). Degradation efficiency and the durability of the as-prepared catalyst to the degradation of MG were assessed in this system. The ZnO/RGO@NF showed a high degradation rate and very stable photocatalytic activity under 5 h continuous operations.

## 2. Experimental Section

### 2.1. Preparationof ZnO/RGO@NFHybrid

The Nickel foams (~320 g/m^2^ and ~1.0 mm thick) were purchased from Shanghai Zhongwei New Material Co. Ltd. (Shanghai, China) and tailored into rectangles shapes (1 cm^2^). All the other chemicals were analytical reagent grade and used without further purification. All of the solutions were prepared with ultrapure water of resistivity 18.2 MΩ cm obtained from a Millipore Milli-Q system. Graphene oxide (GO) was synthesized by the modified Hummers method [36]. The 0.5 mg mL^−1^ GO solution was monitored from a syringe pump (KDS100, Kd Scientific, Holliston, MA, USA) and was fed to a sprayer. The GO spraying process (15 min) was carried out under an air flow (8 L min^−1^) towards the Ni foam, which was placed on a hot plate (80 °C) to enhance/promote the solvent evaporation. After spaying, the GO coated Ni foam (GO@NF) was dried in the air overnight [35].

ZnO nanorods were prepared via a simple two-step process. Firstly, GO@NF was heated in nitrogen at 200 °C for 30 min. The heat-treated GO@NF was immersed into a prepared mixture solution of 0.1 M zinc acetate and 0.1 M hexamethylenetetramine for 20 s, taken out, and then heated at 200 °C for 10 min under nitrogen stream. In order to obtain a more uniform ZnO seed layer, this process was repeated three times. During this thermal process, GO was reduced to RGO, and ZnO seeds were grown on the 3D RGO@NF in one step. Secondly, a hydrothermal process was used for the growth of ZnO nanorods on the seed layer. After washing the ZnO seeds with ultrapure water, the as prepared sample was placed in a 50 mL Teflon-Lined stainless steel autoclave with a colloid solution of 0.2 M zinc acetate (20 mL) and 5 M ammonia solution (3 mL). The autoclave was sealed and heated to 90 °C for 5 h. Finally, the sample was rinsed with ultrapure water and dried at room temperature. The weight proportion of ZnO: RGO: NF in the final hybrid is 1:1.5:18. For control comparison, pure ZnO@NF was also prepared under the same procedure and identical conditions without the GO spraying process. RGO@NF was also prepared without immersing in the mixture zinc solutions.

### 2.2. Physical Characterization

The crystalline properties and morphologies of the as-prepared materials were characterized by powder X-ray diffraction (XRD, D8-advanced, Bruker, Karlsruhe, Germany, 40 kV, 20 mA, Cu Kαradiation), scanning electron microscopy (SEM, JEOL, Tokyo, Japan, JSM6700F) equipped with an X-ray energy dispersive spectrometer (EDS), and transmission electron microscopy (TEM, JEOL-2010, voltage of 200 kV). The atomic composition of the hybrids was detected by X-ray photoelectron spectroscopy (XPS, Perkin Elmer, Waltham Mass, MA, USA, Al Kradiation). Raman spectroscopy was performed using a Jobin-Yvon Lab Ram HR800 system. The solid samples UV-Vis spectra were recorded by UV-Visible Spectrometer (Thermo SCIENTIFIC, Waltham, MA, USA, EVLUTION 220).

### 2.3. Photocatalytic Activities Measurements

The photocatalytic activities of the hybrids were investigated using Methylene blue (MB), Rhodamine (RhB), and mixed MB/RhB as pollutants. Briefly, for every measurement, one slice of the as-prepared hybrid (1 cm^2^) was suspended in 20 mL of dye solution (10 mg L^−1^). Prior to the irradiation, the suspensions were magnetically stirred in the dark for 30 min in order to reach the absorption–desorption equilibrium. The photo degradation process was carried out at room temperature in a reactor equipped with a water jacket that can be used to maintain the operating temperature by water circulation. A 500 W Xe was equipped as a light source to provide UV irradiation. The dye concentration was analyzed by UV-Vis adsorption spectra, using a UV 1800 PC spectrophotometer. The degradation efficiency of dye pollutant was calculated using the dependence: (C_o_ − C/C_o_) × 100%, where C_o_ and C are initial concentration of the dye solution before turning on the illumination and the residual concentration of the dye solution after illumination for the selected time interval. Each experimental point was an average of three independent adsorption tests.

### 2.4. PracticalApplication in Seawater

To test the practical photocatalytic performance of the as-prepared photocatalyst, we repeated the photocatalytic degradation experiments of MG (20 mg/L) in seawater (pH 8.15, conductivity 30.52 mS, after filtration) by the same method that is described above. The durability of the as-prepared photocatalyst was tested in a simple laboratory flow system (shown in Scheme 1). The polluted water sample was prepared by dissolving MG in seawater (10 mg L^−1^). The continuous flow system was set up using three glass columns (an internal diameter of 0.75 cm and a total height of 30 cm) filled with two pieces of chopped ZnO/RGO@NF (1 cm^2^) in each glass column on the top of a supporting layer of glass fiber. A fine glass fiber mesh is placed at a distance of 5 cm from the bottom in order to prevent any loss of the catalysts. The columns were operated by injections of the polluted seawater by a peristaltic pump (Heidolph Co., Schwabach, Germany). The flow rate of the influent was set as 15 mL min^−1^. A Zolix Omni-*λ*150 monochromator (Beijing, China) equipped with a 150 W xenon lamp was used as a light source. The distance between the photo reactor and the light source was 25 cm.

## 3. Results and Discussion

### 3.1. Characterization of Materials

**Figure 1:**
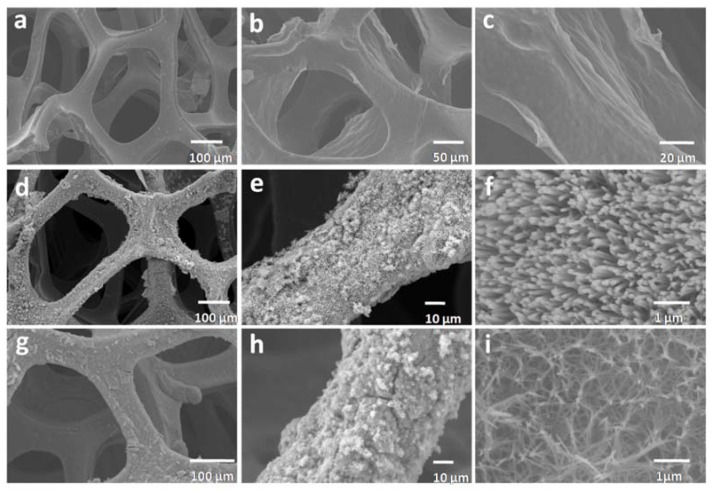
SEM images of Ni foam (**a**), reduced graphene oxide (RGO)@NF (**b**,**c**), ZnO@NF (**d**–**f**), and ZnO/RGO@NF (**g**–**i**).

The SEM images of the pure NF, RGO@NF, ZnO@NF, and ZnO/RGO@NF are shown in Figure 1a–i, respectively, providing insights into the morphology and detailed structure of the as-prepared ZnO/RGO@NF hybrid. First, as shown in Figure 1b, the RGO film has been successfully deposited onto the smooth Ni foam surface displaying a crumpled and curly surface. A panoramic view of the as-prepared hybrid samples shows uniform hierarchical nanorods structures that are made from vertically aligned and hexagonal ZnO nanorods (Figure 1d,e). While the morphology of ZnO nanorods deposited onto RGO-coated NF (RGO@NF) displays interconnected hierarchical networks that are much smaller in diameter (Figure 1h,i). The smaller and thinner of ZnO nanorods is much more desirable, as it could contribute to an increased specific surface area. This phenomenon could be attributed to the step of NF surface treatment, the pre-coating of RGO onto NF, and can be consistent with similar phenomena reported previously. For example, Cui’s group reported that the particle size of TiO_2_ grown on graphene is much smaller than that grown on quartz [37]. The morphology of Au film was affected by graphene layers in Sun’s work [38]. Therefore, it is reasonable to attribute the smaller size of ZnO nanorods to the presence of RGO, which act as templates and seeds layer for the growth of ZnO nanorods. The EDS elemental mapping analysis suggests the presence of Zn, Ni, C, and O elements in the hybrid (Supplementary materials Figure S1).

**Figure 2:**
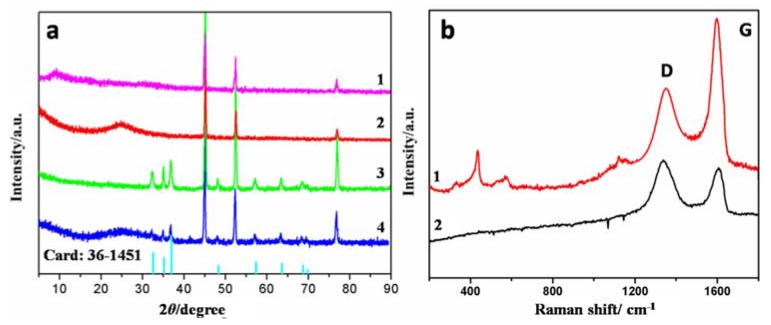
(**a**) XRD of graphene oxide (GO)/NF (curve 1), GO/NF (curve 2), ZnO@NF (curve 3) and ZnO/RGO@NF (curve 4); and (**b**) Raman of ZnO/RGO@NF (curve 1) and GO (curve 2).

Typical XRD patterns of GO@NF, RGO@NF, ZnO@NF, and ZnO/RGO@NF are presented in Figure 2a. Three strong diffraction peaks were observed at 2θ = 44.2°, 51.6°, 76.1° in all of the samples, which correspond to (111), (200), and (220) planes of Ni foam, respectively [39]. The GO@NF sample displayed a typical characteristic peak at 2*θ* = 9.2°, which is attributed to the (002) reflection of graphene oxide. The peak shifted to 2*θ* = 24.5° after the thermal treatment (i.e., RGO@NF), corresponding to a smaller interlayer space between graphene sheets from 0.95 to 0.36 nm, according to Bragg’s law (Supplementary materials Equation S1). This is because the functional groups on graphene sheets were eliminated and indicate the successful reduction of GO in the hybrid sample [40]. In addition, the peaks of 2*θ* values in the ZnO@NF and ZnO/RGO@NF samples at 31.8, 34.6, 36.4, 47.7, 56.7, 63.0, 67.7 and 68.8 can be indexed to the (100), (002), (101), (102), (110), (103), (112), and (201) crystal planes of hexagonal wurzite phase of the ZnO structure, with a space group of P6_3_mc in accordance with the JCPDS Card No. 36-1451 [41]. Raman spectroscopy is a useful technique for the determination of *sp*^2^ and *sp*^3^ hybridized carbon atoms in graphene. In GO spectrum, specific *D* band (at 1350 cm^−1^) and *G* band (at 1605 cm^−1^) were observed, which originated from the out–plane vibration of *sp*^3^ carbons (representing defects) and the in plane vibration of *sp*^2^ carbons in the aromatic conjugated system, respectively. The high intensity ratio of *D* to *G* bands (*I_D_*/*I_G_*) of 1.81 indicates the presence of many defects, which are in the form of epoxide and other oxygen carrying groups. This ratio reduced to 1.02 in ZnO/RGO@NF, suggesting the removal of oxygen carrying functionalities and successful reduction of GO to RGO [42]. Meanwhile, the two bands at 437.5 and 582.8 cm^−1^ are the signature peaks of ZnO (corresponding to *E*_2_ and *E*_1_ modes of crystalline ZnO, respectively), showing the existence of ZnO in ZnO/RGO@NF [43]. Therefore, both XRD and Raman measurements confirm the successful synthesis of ZnO/RGO@NF composite.

**Figure 3:**
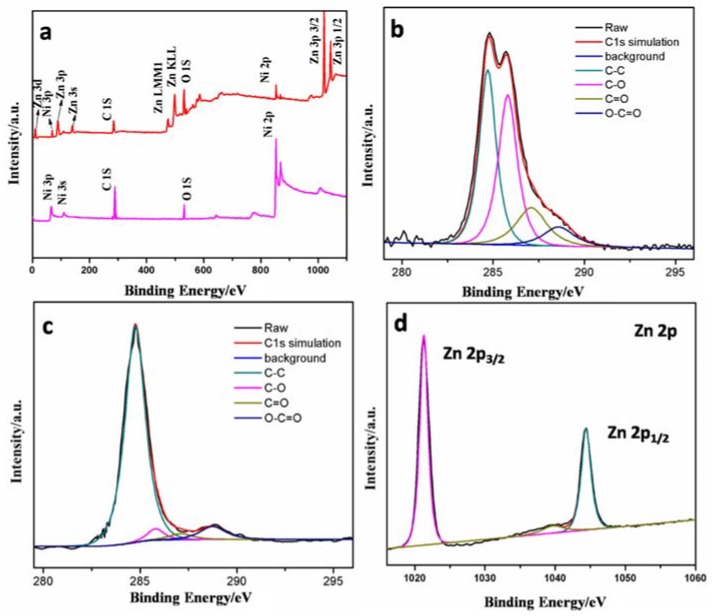
(**a**) X-ray photoelectron spectroscopy(XPS) survey spectra of GO/NF and ZnO/RGO@NF; C1s XPS for GO (**b**) and ZnO/RGO@NF (**c**); and, (**d**) XPS survey spectra of Zn.

To investigate the chemical states of elements in the hybrids, the wide-scan XPS was carried out and the results are shown in Figure 3a. Characteristic signal of Zn*2p* is found at 1020 eV in the sample of ZnO/RGO@NF besides the signals of C*1s*, O*1s,* and Ni*2p* observed in GO/NF sample, indicating the successful deposition of ZnO on 3D graphene foam. The C*1s* XPS spectrum of GO (Figure 3b) could be deconvoluted into four peaks arising from C-C/C=C (284.6 eV) in the aromatic rings, C-O (285.8 eV) of epoxy and alkoxy, C=O (287.1 eV) and O-C=O (288.8 eV) groups, respectively [44]. The intensity of the oxygenated groups decreases significantly in ZnO/RGO@NF (Figure 3c), indicating that GO was reduced to RGO during the thermal process. This result is in a good agreement with the results that were obtained from XRD and Raman. The Zn*2p* XPS spectrum is also given in Figure 3d, the peaks at 1021 eV (for Zn*2p*_3/2_*)* and 1044 eV (for Zn*2p*_1/2_) further confirmed the presence of ZnO in the composite.

### 3.2. Characterization of Photocatalytic Activity

**Figure 4:**
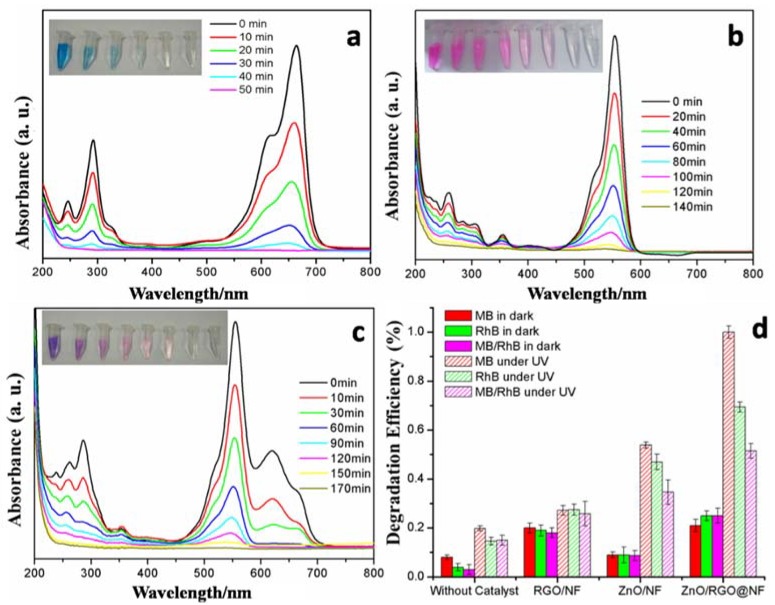
(**a**–**c**) The photocatalytic degradation process of MB (10 mg/L), RhB (10 mg/L), MB/RhB (5 mg/L/5 mg/L) mixed solutions in the presence of ZnO/RGO@NF under UV irradiation (the inset photos show the color change of different dyes solution with time); (**d**) The degradation efficiency of MB (10 mg/L), RhB (10 mg/L), and MB/RhB (5 mg/L/5 mg/L) mixed solution in the presence of different photocatalysts under UV light irradiation.

The outlets from the industries always contain a variety of organic dyes, which make the purification of waste water difficult. To verify the photocatalytic activity of the as-prepared hybrid to different dyes, methylene blue, rhodamine B (RhB), and the mixed solution of methylene blue and rhodamine B were used as model pollutants to simulate real waste water from industry. Figure 4a–c exhibits the gradual photodegradation process of MB, RhB, and mixed MB/RhB in the presence of the ZnO/RGO@NF catalyst under UV light irradiation. The removal efficiency of MB, RhB, and mixed MB/RhB in dark and under UV light irradiation by different photocatalysts were compared in Figure 4d. The absorption abilities of the as-prepared catalysts were tested after the system was stirred for one hour under dark. The sample of RGO@NF and ZnO/RGO@NF could remove much higher dye pollutants (about 20%) than the ZnO@NF sample (only 10%) (Figure 4d). This suggests that the existence of RGO in the hybrid promotes the higher efficiency of the pollutant adsorption. It is believed that the effective adsorption of target molecules near the metal oxide photocatalytic surface can accelerate the photoexcited electron transfer, facilitating the photocatalytic reaction [28]. The ZnO/RGO@NF hybrid demonstrated the highest removal efficiency for the three dye pollutants after being exposed under UV light for one hour. It degraded 100% of MB, 69.4% of RhB, and 51.5% of MB/RhB, while only 53.8%, 47.0%, and 34.7% of these pollutes were degraded by ZnO/NF, respectively. The degradation efficiency of the pollutants was lower for the photocatalyst of RGO/NF, with only 27.2%, 27.5%, and 25.8% of MB, RhB, and MB/RhB degraded, respectively.

The high photocatalytic efficiency of ZnO/RGO@NF can be related to a more efficient photo excited electron transfer from the ZnO to the RGO [45,46]. The effective transfer of the emitted electrons from the conduction band of ZnO to graphene suppresses the generated electron-hole recombination on the ZnO surface and generates more photo-electrons. Trapped electrons react with oxygen to form superoxide radicals, whereas the holes in the valence band of ZnO react with water to form hydroxyl radicals. The hydroxyl radicals can convert to H_2_O_2_ and superoxide radical anions to regenerate hydroxyl radicals. Finally, the derived hydroxyl radicals decompose the MB and RhB to carbon dioxides and water. In addition, the ZnO nanorod in ZnO/RGO@NF has a smaller size in diameter than it is in ZnO@NF, which can offer a more photo-active surface. Furthermore, under the effect of RGO, dye molecules can be strongly adsorbed near the ZnO photocatalytic surface and accelerate photoexcited electron transfer.

**Figure 5:**
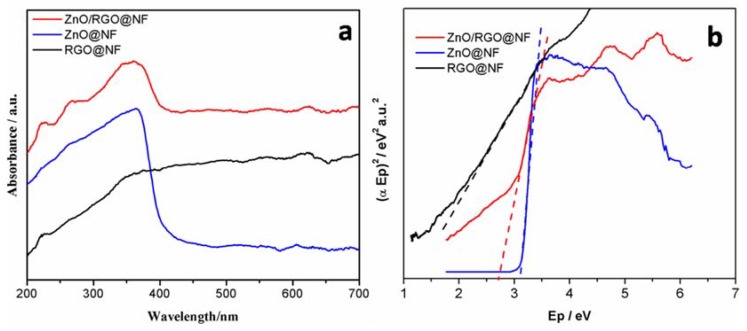
(**a**) UV-vis absorption spectra and (**b**) Tauc plots showing band gap of RGO/NF, ZnO/NF, and ZnO/RGO@NF.

The UV-vis absorption spectra of ZnO/RGO@NF, ZnO@NF and RGO@NF (Figure 5a) were investigated, and accordingly the band gaps were calculated by Kubelka-Munk remission function (Figure 5b). With the introduction of RGO in the hybrid, the absorption of ZnO/RGO@NF showed an enhanced absorbance in the visible ranging from 400–700nm. Accordingly, the band gap of ZnO/RGO@NF was reduced to 2.64 eV from 3.16 eV of ZnO@NF [47]. At the same time, this supports the observation of a red shift in the absorption edge of the ZnO/RGO@NF when compared to ZnO@NF. The hybrid of ZnO/RGO@NF is visible light active. Dipti P. Das [46] also reported UV light active “metal oxides” transfiguring to visible light active by RGO. When comparing the degradation efficiency of the hybrids under visible light and UV light, the degradation efficiency of the hybrids for MB is also tested in visible light under the same condition (Figure S2). Under visible light, the ZnO/RGO@NF shows the highest photoactivity (78% degradation in 50 min) and RGO@NF shows the lowest photoactivity (32% degradation in 50 min). When comparing the degradation efficiency of the hybrids, we found under UV light, the dyes are degraded in shorter time. Actually, the similar results have been reported [45]. So, the following experiments are preceded under UV light.

Comparative results of the performance of this fabricated photocatalyst as well as other ZnO-based photocatalyst in organic dye degradation are listed in Table S4. With the loading of ZnO on 3D RGO@NF, the efficiency of the proposed photocatalyst was either comparable with or higher than those provided by other ZnO or ZnO/RGO hybrids.

### 3.3. Application of *ZnO/RGO@NF* as Photocatalytic Catalysts of MG in Seawater

**Figure 6:**
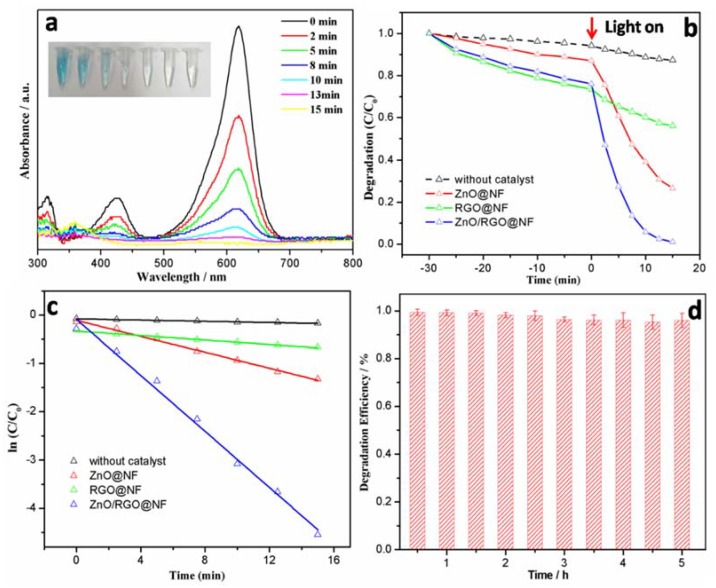
(**a**) The photocatalytic degradation process of malachite green (MG)(20 mg/L) in seawater in the presence of ZnO/RGO@NF under UV irradiation(the inset photo shows the color change of MG in seawater with time); (**b**) MG degradation (%) at different irradiation time and (**c**) first order kinetics plot of ln(C/C_0_) versus irradiation time for MG degradation; and, (**d**) Degradation efficiency of ZnO/RGO@NF for continuous 5 h degradation of MG (10 mg/L) in seawater.

The photocatalytic degradation of MG in seawater was tested. Prior to the light irradiation, the suspensions were magnetically stirred in the dark for 30 min to reach the absorption–desorption equilibrium. As shown in Figure 6a, ZnO/RGO@NF gives the significantly high photocatalytic activity for the degradation of MG in seawater. The photocatalytic degradation of MG quickly reached 100% when exposed under UV light for only 15 min, while only 68% degraded by ZnO@NF, 38% by 3D RGO@NF (Figure 6b). The degradation of MG was analyzed by pseudo-first order kinetics, according to the previous reports [29,47]. The kinetic of ZnO/RGO@NF, ZnO@NF, and RGO@NF efficiency (%) on MG was studied and the degradation rate constants (k) are 0.29, 0.06, and 0.01 min^−1^, respectively (Figure 6c). The rate constant of ZnO/RGO@NF was about 4.8 times higher than that of ZnO@NF.

The practical photocatalytic performance and durability of the as-prepared ZnO/RGO@NF to MG was assessed in a continuous flow seawater system. The video (Supplementary Materials Video S1) shows that, under UV light irradiation, the MG in seawater was efficiently degraded by ZnO/RGO@NF. Effluent from the columns was periodically sampled (30 min) and analyzed by UV–vis spectroscopy. As can be seen in Figure 6d, ZnO/RGO@NF showed stable photocatalytic performance, no significant change was observed for the photocatalytic activity of MG under 5 h continuous operations. In this simple flow seawater system, the polluted seawater was purified 22L/d by 6 cm^2^ 3D ZnO/RGO@Ni foam catalyst. 

## 4. Conclusions

A novel ZnO nanorods modified 3D RGO@NF was synthesized by a simple hydrothermal process, which has been used as photocatalyst for the degradation of organic dyes. With the addition of RGO, the hybrid of ZnO/RGO@NF showed well adsorption capacity in dark. Owing to the synergistic effect of 3D RGO and ZnO nanorods in the hybrid, significantly higher photocatalytic degradation efficiency of ZnO/RGO@NF was observed when compared to RGO@NF and ZnO@NF. The practical photocatalytic performance and the durability of the as-prepared ZnO/RGO@NF to the degradation of MG in seawater were assessed. ZnO/RGO@NF showed high efficiency for the degradation of MG and stable photocatalytic performance for 5 h continuous operation, showing its great potential as catalysts for the efficient removal of MG in seawater.

## Supplementary Materials

The following are available online at http://www.mdpi.com/1996-1944/11/6/1004/s1, Figure S1: EDS spectrum of ZnO/RGO@Ni Foam, Figure S2: (a) The photocatalytic degradation process of MB (10 mg/L) in the presence of ZnO/RGO@NF under visible light irradiation; (b) MB degradation (%) at different irradiation time with or without catalyst under visible light irradiation, Equation (S1): Bragg’s law, Table S1: Comparison of photocatalytic activity of ZnO/RGO based photocatalysts for dyes degradation, Video S1: Under UV light irradiation the malachite green (10 mg L^−1^) in seawater degraded by ZnO/RGO@NF in a continuous flow sea water system.
