# Supplementary material for: Efficient Photocatalytic Degradation of Malachite Green in Seawater by the Hybrid of Zinc-Oxide Nanorods Grown on Three-Dimensional (3D) Reduced Graphene Oxide(RGO)/Ni Foam
Source: Materials (Basel). 2018 Jun 13;11(6):1004. doi: 10.3390/ma11061004 (PMC6025546; doi:10.3390/ma11061004)
Supplement: Supplementary file 1 [file materials-11-01004-s001.zip › materials-308042-supplementary materials/materials-308042-supplementary materials.pdf]

# Supplementary Materials

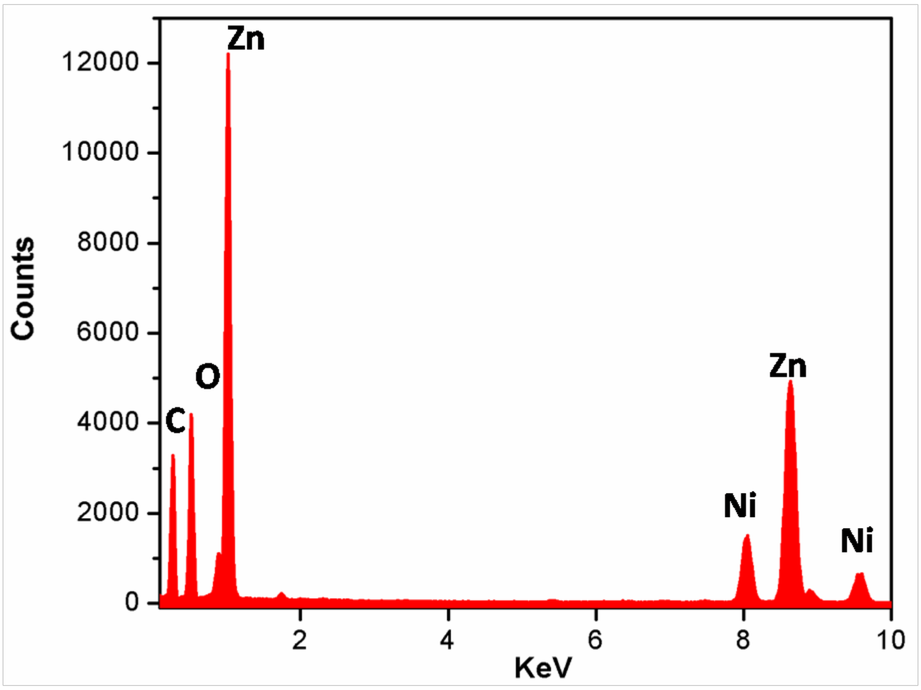


**Figure S1.** EDS spectrum of ZnO/RGO@Ni Foam.

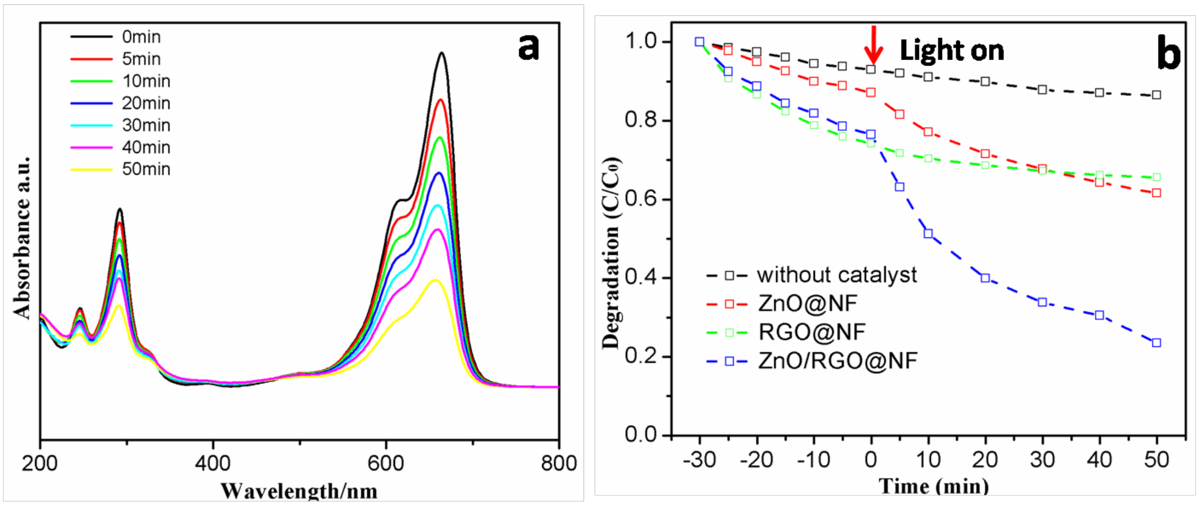


**Figure S2.** (a) The photocatalytic degradation process of MB (10 mg/L) in the presence of ZnO/RGO@NF under visible light irradiation; (b) MB degradation (%) at different irradiation time with or without catalyst under visible light irradiation.

Video S1 shows that under UV light irradiation the malachite green in seawater (10 mg $L^{-1}$) is efficiently degraded by ZnO/RGO@NF in a continuous flow sea water system.

Bragg's law (Equation S1)

$$n\lambda=2d\sin\theta$$

n: order of diffraction; $\lambda$: incident X- ray wave length; $\theta$: the Bragg angle in degrees (diffraction angle); $d$: the spacing between lattice planes.

**Table S1.** Comparison of photocatalytic activity of ZnO/RGO based photocatalysts for dyes degradation.

| Photocatalyst | Dye | Dye Con (mg/L) | Catalyst load (g/L) | Irradiation time (min) | degradation | Ref |
|---|---|---|---|---|---|---|
| ZnO/RGO nanocomposite | Methyl orange | 10 | 1.00 | 60 min | 80% | [1] |
| Ni-ZnO/ RGO/nylon-6/Ag | Methyl orange | 5 | 1.00 | 30 min | 92% | [2] |
| Uniform ZnO/RGO | MB | 10 | 0.50 | 120min | 60% | [3] |
| ZnO microspheres/RGO | MB | 10 | 1.00 | 20 min | | [4] |
| Fe3O4-ZnO core/shell nanoparticles/RGO | MB | 10 | 0.30 | 120 min | 96% | [5] |
| Nano-ZnO/RGO | Neutral red | / | 0.40 | 20 min | 100% | [6] |
| | Crystal violet | | | 80 min | 97% | |
| | Congo red | | | 150 min | 68% | |
| | Methyl orange | | | 150 min | 66% | |
| ZnO/RGO@NF | MB | 10 | 0.25 | 50 min | 100% | This work |
| | RhB | 10 | | 140 min | 100% | |
| | MB and RhB | 5/5 | | 170 min | 100% | |
| | MG | 20 | | 30 min | 100% | |


1. Chin, B.O.; Abdul, W.M.; Law, Y.N.; Ebrahim, M.; Sepehr, A.; Nur, H.H.H. Solar photocatalytic and surface enhancement of ZnO/rGO nanocomposite: Degradation of perfluorooctanoic acid and dye. *Process Saf. Environ. Prot.* **2017**, *112*, 298–307, doi:10.1016/j.psep.2017.04.031.
2. Roya, S.; Mastaneh, S.; Mohammad, R.Z.; Ali, A.S. High-performance visible light-driven Ni-ZnO/rGO/nylon-6 & Ni-ZnO/rGO/nylon-6/Ag nanofiber webs for degrading dye pollutant and study their antibacterial properties. *J. Alloys Compd.* **2017**, *729*, 921–928, doi:10.1016/j.jallcom.2017.09.173.
3. Xue, B.; Zou, Y. Uniform distribution of ZnO nanoparticles on the surface of grpahene and its enhanced photocatalytic performance. *Appl. Surf. Sci.* **2018**, *440*, 1123–1129, doi:10.1016/j.apsusc.2018.01.299.
4. Qin, J.; Zhang, X.; Yang, C.; Cao, M.; Ma, M.; Liu, R. ZnO microspheres-reduced graphene oxide nanocomposite for photocatalytic degradation of methylene blue dye. *Appl. Surf. Sci.* **2017**, *392*, 196–203, doi:10.1016/j.apsusc.2016.09.043.
5. Ghanbarnezhad, S.; Baghshahi, S.; Nemati, A.; Mahmoodi, M. Preparation, magnetic properties, and photocatalytic performance under natural daylight irradiation of Fe3O4-ZnO core/shell nanoparticles designed on reduced GO platelet. *Mater. Sci. Semicond. Process.* **2017**, *72*, 85–92, doi:10.1016/j.mssp.2017.09.015
6. Raji, A.; Thomas, N.J.I.E.; Suguna, P.; Mani, S.; Yong, R.L. Direct solvothermal synthesis of zinc oxide nanoparticle decorated graphene oxide nanocomposite for efficient photodegradation of azo-dyes. *J. Photochem. Photobiol. A* **2017**, *337*, 100–111.


© 2018 by the authors. Submitted for possible open access publication under the terms and conditions of the Creative Commons Attribution (CC BY) license (http://creativecommons.org/licenses/by/4.0/).
